# Determinants of Neonatal and Postneonatal Mortality in Northeastern Brazil: A Cohort Study of Newborns Admitted to the Neonatal Intensive Care Unit

**DOI:** 10.3390/healthcare12131249

**Published:** 2024-06-23

**Authors:** Maria Goretti Policarpo Barreto, Cláudia Silva, Renata Policarpo Barreto, Roberta Policarpo Barreto, Lara Moreira Teles de Vasconcelos, Maria Conceição Manso

**Affiliations:** 1 Faculdade de Ciências e Tecnologia Universidade Fernando Pessoa, Praça 9 de Abril 349, 4249-004 Porto, Portugal; 2 Hospital Regional Unimed Fortaleza (HRU), Avenida Visconde do Rio Branco, 400, São João do Tauape, Fortaleza 60420-570, CE, Brazil; rpolicarpobarreto@gmail.com (R.P.B.); isis_roberta@yahoo.com.br (R.P.B.); laramtvasconcelos@gmail.com (L.M.T.d.V.); 3 Faculdade de Ciências da Saúde, RISE-Health, Universidade Fernando Pessoa, Rua Carlos da Maia, 296, 4200-150 Porto, Portugal; csilva@ufp.edu.pt; 4 FP-I3ID, FP-BHS, Universidade Fernando Pessoa, Praça de 9 de Abril 349, 4249-004 Porto, Portugal; 5 Centro de Ciências da Saúde, Universidade de Fortaleza (UNIFOR), Avenida Washington Soares, 1321, Edson Queiroz, Fortaleza 60811-905, CE, Brazil; 6 Centro de Ciências da Saúde, Universidade Federal do Ceará (UFC), Avenida da Universidade, 2853, Benfica, Fortaleza 60020-181, CE, Brazil; 7 REQUIMTE-LAQV (Laboratório Associado de Química Verde), Universidade do Porto, 4050-313 Porto, Portugal

**Keywords:** child mortality rate, neonatal intensive care, neonatal mortality, risk factors, sanitation

## Abstract

Despite advances in neonatology, neonatal mortality from preventable causes remains high in the North and Northeast regions of Brazil. This study aimed to analyze the determinants associated with neonatal and postneonatal mortality in newborns admitted to a neonatal intensive care unit. A cohort study was carried out in a capital in the Brazilian Northeast from 2013 to 2018. The outcome studied was death. Poisson regression was performed in the multivariate analysis of variables. Four hundred and eighty newborns were eligible, and 8.1% (39 newborns) died. Among them, 34 died in the neonatal period. The determinants that remained significantly associated with neonatal and postneonatal mortality in the final adjustment model (*p* < 0.05) were history of abortion, perinatal asphyxia, early neonatal sepsis and umbilical venous catheterization. All causes of this outcome were preventable. The neonatal mortality rate, although it did not include twins, neonates with malformations incompatible with life and other conditions, was 3.47 deaths per thousand live births (95% CI:1.10−8.03‰), well below the national average. In this study, pregnant women from different social classes had in common a private plan for direct access to health services, which provided them with excellent care throughout pregnancy and postnatal care. These results indicate that reducing neonatal mortality is possible through public policies with strategies that promote improvements in access to health services.

## 1. Introduction

The Child Mortality Rate (CMR) integrates the neonatal and postneonatal components [1] and is a classic indicator of a country’s health, which assesses the health situation and health care, reflecting on children’s quality of life and care provided [2].

The world’s CMR was reduced by more than 50% between 1990 and 2017 (falling from 12.6 million to 5.4 million children, respectively). The CMR has still been high in developing countries, especially in the neonatal period [3]. The Early Neonatal Mortality Rate (ENMR) is an index that reflects the socioeconomic conditions of a region and the quality of medical care provided in prenatal care, childbirth and the newborn after delivery [2]. From 1990 to 2019, the worldwide Neonatal Mortality Rate (NMR) was reduced by 55.2%, from 38 to 17 deaths per thousand live births (LBs). In 2019, about 2.4 million children died in the first life month, corresponding to 6700 neonatal deaths per day [3].

Studies had reported that prematurity and low birth weight were the risk factors that most affect neonatal morbimortality [4,5] by consequences such as necrotizing enterocolitis, perinatal asphyxia and respiratory distress syndrome [5].

A Brazilian population-based study revealed that the NMR was still high, accounting for approximately 40% of neonatal deaths. Those were associated with perinatal asphyxia in babies within their best birth weight and without malformations, from low-risk pregnancies [6].

Two Brazilian geographic regions—Northeast and North—had the highest CMRs due to poor access to health care and environmental conditions [7,8]. In 2018, a study performed comparing CMRs between isolated states found a CMR of 9.5 in the State of Santa Catarina (South region) compared with a rate of 22.6 in the State of Amapá (North region). In the same year, the State of Ceará (Northeast region) had a CMR of 13.4, closer to the Brazilian national rate of 13.1, due to improved sanitary conditions. In less than three decades, Ceará reduced the CMR from 25.5 deaths per thousand LBs in 1990 to 9.5 deaths per thousand LBs in 2018, slightly higher than the national average (9.1 deaths per thousand LBs). Although the situation had improved, the number of preventable deaths was still high [9].

To help reduce the CMR, a major investment in sanitation infrastructure is needed in Brazil. This will be reflected in the improvement of public health [10].

Sanitation is relevant to the population’s quality of life. The lack of an adequate garbage collection and sanitary sewage system contributes to the proliferation of waterborne diseases and contributes to the degradation of water resources, increasing infant morbimortality [11].

In 2019, the “Continuous National Household Sample Survey” showed relevant disparities in basic sanitation in different Brazilian regions. In the North region, access to the sewage network was available in 27.4% of households. In the Northeast, coverage of this service was 47.2%. The Southeast region had 88.9% coverage, while the South and Central-West regions accounted for 68.7% and 60% of households, respectively. The first regions need adequate investments to improve sewage infrastructure [12].

In recent years, several publications have approached population morbimortality, whether at a regional, national or international level, as referenced previously. There are few articles that discuss this topic contextualized in supplementary health services. Most of the current literature refers to research in public institutions, denoting the lack of published data in the private sector [4,6,7,8,9,13,14].

The present study aims to evaluate the environmental, maternal and neonatal determinants associated with neonatal and postneonatal mortality in newborns admitted to neonatal intensive care units in private hospital.

This research presents relevant epidemiological results for decision-making in strategic planning, aiming to improve services for pregnant women and children and expand assistance to pregnant women in prenatal and childbirth and to the newborn to reduce mortality rates.

## 2. Materials and Methods

### 2.1. Study Design, Location and Period

A cohort study was carried out with all the newborns admitted to the Neonatal Intensive Care Unit (NICU) of a tertiary hospital in the private sector who met the eligibility criteria, carried out over a period of six years, between January 2013 and December 2018, in the city of Fortaleza, capital of the state of Ceará, located in northeastern Brazil.

The Hospital Regional Unimed, where the authors conducted this study, was administrated by one of the major health care corporations in Brazil and was a reference hospital on a state and a national level for high-risk pregnancies.

### 2.2. Participants and Eligibility Criteria

This study considered all newborns born to mothers living in Fortaleza, born alive in the obstetric center of the aforementioned institution, who required hospitalization in the NICU after birth, followed up until discharge or death. Twins, neonates with malformations that were incompatible with life and babies that were transferred to and from other hospitals or that were readmitted at the NICU were excluded from this study. Regarding twins, multiple gestation is rare, accounting for 2 to 4% of all births, with a prevalence ranging from 0.9 to 2.4% in Brazil, and is associated with worse maternal and perinatal outcomes. Twin pregnancies are strongly associated with severe maternal morbidity, maternal near-misses and perinatal morbidity, especially for the second twin [15]. For this reason, twin pregnancies were excluded from our study.

### 2.3. Dependent Variable

Death was considered a dependent variable. Death was classified [16] within the periods that occurred as early neonatal death (less than 7 days old), late neonatal death (between 7 and 28 days old) and postneonatal death (more than 28 days old).

### 2.4. Independent Variables

The independent variables were split into environmental, maternal and newborn factors.

#### 2.4.1. The Environmental Factors

The environmental factors were hydro-sanitary support, such as the access to potable water and a sewage system integrated with residences and residential locations based on neighborhood and city section.

#### 2.4.2. The Maternal Factors

The maternal factors were the sociodemographic profiles of mothers and characteristics of gestation and of birth. The maternal sociodemographic variables included age, marital status and education. The gestational variables included access to prenatal care, number of prenatal consultations; number of pregnancies and deliveries (parity); abortion history; antenatal corticosteroid use for gestational age (GA) between 24 and 36 weeks; and presentation (cephalic or pelvic/feet/breech) and clinical complications (comorbidities) during gestation; such as hypertensive syndromes (high blood pressure, preeclampsia, eclampsia and HELLP syndrome), gestational diabetes, third-trimester bleeding, thrombophilia, infectious diseases [known as TORCHS (toxoplasmosis, rubella, cytomegalovirus, herpes simplex and syphilis) and urinary tract infection], placental abruption and placenta previa.

The variables related to delivery and complications in labor were delivery type (cesarean or vaginal), umbilical cord prolapse, abnormal bleeding, meconial amniotic fluid, shoulder distortion and fetal distress.

#### 2.4.3. The Newborns’ Factors

The newborns’ variables were sex, GA (in weeks), birth weight and adequacy for GA, Apgar score (divided into anoxia in Apgar scores <7 and no anoxia in Apgar scores of 7–10 [17]), the need for neonatal resuscitation, clinical complications (metabolic disorders, neonatal anoxia, respiratory distress syndrome, patent ductus arteriosus and early neonatal sepsis (occurs in the first 72 h of life) or late neonatal sepsis (occurs after the first 72 h of life), diagnosed by clinical or laboratorial criteria) and therapeutical interventions (oxygen support, ventilatory assistance and central line).

GA was determined by best estimate, using either the date of the last menstrual period or early ultrasound, or by physical examination of the newborn (using the Capurro somatic or New Ballard method). The newborns were classified as premature when born before the 37th week of gestation. Birth weight measured in grams allowed categorization of the neonates with severely low birth weight (<1500 g) and low (≥1500 g) or normal birth weight (≥ 2500 g).

The newborns were also classified according to nutritional state, following the World Health Organization’s intrauterine growth curves, as adequate for GA (when birth weight was between the 10th and 90th percentiles), small for GA (below the 10th percentile) and large for GA (above the 90th percentile).

The Apgar score was evaluated in the first and fifth minutes of life. The babies were divided into two categories: without perinatal anoxia (Apgar ≥7) and having undergone perinatal anoxia (Apgar < 7).

#### 2.4.4. Data Gathering

The data were collected from the medical records of mothers and newborns. Data regarding access to sanitation and treated water were obtained from the local water and sewage management company.

### 2.5. Statistical Analysis

Statistical analysis was performed on IBM SPSS Statistics vs. 25.0^®^ (IBM Corp., Armonk, NY, USA). All inference procedures were performed with a significance level of 0.05.

The ENMR, the late neonatal mortality rate (LNMR) and the postneonatal mortality rate (PNMR) in this cohort were estimated. Each mortality rate was obtained by dividing the number of deaths within an age group by the number of births in a specific location and expressed per thousand LBs. Their confidence intervals were estimated according to the Exact method.

The qualitative variables were described using counts and their corresponding percentages while the quantitative variables were described using means and standard deviations (to allow for comparison with published data).

Initially, the variables were described as simple estimates. To identify factors associated with the outcome of death, the qualitative variables were analyzed by the Pearson’s chi-square test or a Fisher test. For the quantitative variables, bivariate analysis was performed using the Mann–Whitney test (due to non-normal distribution). In the construction of the multivariate analysis, Poisson’s regression with a robust variant and Wald’s statistics were performed.

**Figure 1:**
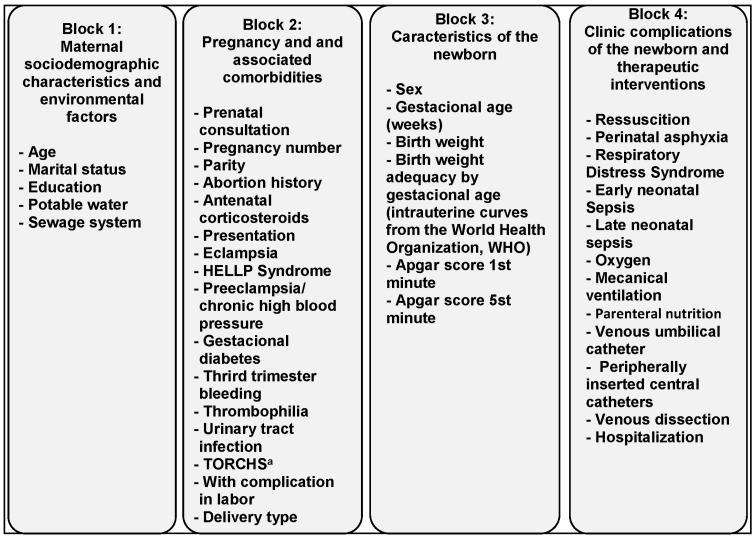
Theoretical model of variables distributed in four blocks for statistical analysis—1st step. Source: authors. TORCHS^a^ = toxoplasmosis, rubella, cytomegalovirus, herpes simplex and syphilis. Source: authors.

The option for an analytical model in two stages allowed proper identification of the risk factors associated with the outcome of “death”. The multivariate analysis, using Poisson’s regression model, was guided firstly by a theoretical model (Figure 1) built for the present research and in which the independent variables were grouped into four blocks: 1—Maternal sociodemographic characteristics and environmental factors; 2—Pregnancy and associated comorbidities; 3—Characteristics of the newborn; and 4—Clinical complications of the newborn and therapeutic interventions. In the first stage of the multivariate model, variables whose bivariate analyses showed significant associations at significance levels of 15% (*p* = 0.15) were introduced. The final model was adjusted with all the variables that, in the first stage and in each block, had significance levels of 5%. The collinearity effects between the explanatory variables were analyzed. In the case of collinearity, only one of the variables remained in the model.

As a measure of the association between the factors of interest and the deaths of newborns, point estimates of the relative risk (RR) and respective confidence intervals (CIs) were calculated.

### 2.6. Ethical Aspects

This study followed all the ethical precepts that regulate research involving human beings, respecting Resolution of the National Health Council Nº. 466/12, submitted to PLATAFORMA BRASIL (CAAE: 92116818.8.0000.8307) and approved by the Research Ethics Committee of the University of Fortaleza—UNIFOR (protocol code 2.753.240).

## 3. Results

In this hospital and during the study period, there were 9778 children that were born alive. Among those, 894 (9.1%) were admitted to the NICU, forming the total population of this study. The sample consisted of 480 neonates who fulfilled the eligibility criteria. A total of 414 neonates of the population (46.3%) were excluded according to the exclusion criteria (259 newborns whose mothers did not live in Fortaleza, 151 newborns from multiple pregnancies, 2 newborns with severe malformations incompatible with life and 2 newborns transferred to other institutions).

In this cohort, thirty-nine deaths (8.1%) were identified: 3.99 deaths per thousand LBs (95% CI = 1.19–8.26 deaths per thousand LBs). Among these 39 newborn deaths, 23 (59.0%) occurred within early neonatal age (2.35 deaths per thousand LBs (95% CI = 0.95–7.23 deaths per thousand LBs)), 11 (28.2%) were late neonatal deaths (1.12 deaths per thousand LBs (95% CI = 0.08–5.08 deaths per thousand LBs)) and 5 (12.8%) were postneonatal deaths (0.51 deaths per thousand LBs (95% CI = 0.05–2.25 deaths per thousand LBs)). Then, this cohort’s neonatal mortality rate (ENMR and LNMR) was 3.47 deaths per thousand LBs (95% CI = 1.10–8.03 deaths per thousand LBs), and the PNMR was 0.51 deaths per thousand LBs.

Table 1 shows the analysis of the variables from blocks 1 and 2 and Table 2 from blocks 3 and 4, following the model suggested in Figure 1. These two tables describe the characterization of the population by point estimates and the distribution of variables according to the presence of the death outcome (bivariate analysis).

Bivariate analysis showed that maternal previous abortion history, complications during birth (Table 1), newborns with average GAs of 30 weeks, neonates with birth weights <1500 g, 1st-minute Apgar scores ≤ 3, 5th-minute Apgar scores < 7, neonatal resuscitation, mechanical ventilation, parenteral nutrition, venous umbilical catheterization, venous dissection, perinatal asphyxia, respiratory distress syndrome and early neonatal sepsis had statistically significant associations with death (Table 2).

Table 3 summarizes the bivariate and multivariate analyses through which the final model shows the joint effect of the evaluated variables that were statistically significantly related to the neonatal deaths that occurred in this cohort of 480 neonates. These were neonates whose mothers had abortion histories in previous pregnancies (*p* = 0.047; RR = 1.774 (95% CI = 1.007–3.126)), that underwent neonatal anoxia (*p* = 0.001; RR = 2.669 (95% CI = 1.48–4.813)), that underwent venous umbilical catheterization (*p* = 0.021; RR = 3.275 (95% CI = 1.199–8.948)) and that had early neonatal sepsis (*p* = 0.004; RR = 6.843 (95% CI = 1.824–25.675)).

## 4. Discussion

In this study’s final model, there were statistically significant associations with abortion history, perinatal asphyxia, early neonatal sepsis and venous umbilical catheterization, and these variables are considered determinants for neonatal and postneonatal mortality. Analysis of these determinants related to mortality rate was essential for the development of effective individualized public policies for each location, whether at the municipal, state or national level, both in the private and public sectors [4].

The neonatal mortality rate was 3.47 deaths per thousand LBs in this cohort (95% CI = 1.10–8.03 per thousand LBs). Considering early neonatal mortality (in the first week of life), there were twenty-three deaths, which represented more than two-thirds of the neonatal deaths. Similar results were also found in other studies [18,19], although the first was an ecological type of study and for the second, the exclusion criteria were neonates with birth weights of fewer than 500 g or born before 24 gestational weeks.

One of this study’s limitations was the lack of data that could specify the causes of maternal miscarriage. Previous abortion history was significantly associated with negative outcomes, showing a risk of neonatal death of 77% for neonates whose mothers had previous abortion histories, confirming the literature data [20,21,22].

Another limitation was the restricted location, only including neonates whom mothers reside in Fortaleza city. In addition, mortality was low in the NICU. Consequently, the number of deaths in the studied population was very small, which did not allow the stratification of these deaths in the analysis of associations. This limitation had a great impact on the number of parturients included in this study in relation to the total LBs in the period from 2013 to 2018 in Fortaleza (224,531) and in Ceará (771,607) [23]. Enhancing the period of this study might show associations between some risk factors not yet identified, but it might have significant associations with neonatal and postneonatal deaths.

Among the maternal factors related to the sociodemographic characteristics analyzed, non-statistically significant association was observed between maternal age, marital status, education and access to basic sanitation (sewage network and treated water) and neonatal and postneonatal death. Similar results were found in other studies [1,4,5,18,19].

This study considered that the non-statistical association between the maternal sociodemographic characteristics and neonatal death was because the newborns remained hospitalized since birth and were not exposed to the socio-environmental factors of a community [19]. In this research, deaths were more directly related to newborn care rather than social conditions, indicating that sociodemographic conditions help to determine the quality of care to this population. Only 51.5% of the mothers lived in residence with access to sewage. These data were not significantly associated with neonatal deaths, but they are still an indication of the need to improve basic sanitation in the city.

The antenatal use of corticosteroids, as a protective factor against neonatal mortality, is a universal recommendation for pregnant women at risk of preterm delivery between the 24th and 36th weeks of gestation. The steroids are used to mature the fetus’ lungs [24,25]. Some Brazilian studies have shown major differences in the use of antenatal steroids, with rates ranging from 48.6% to 81.1% [26,27]. In this casuistry, a low rate of antenatal corticosteroid administration (41.2%) was demonstrated.

Delivery complications were excluded from the final analysis after adjustment of variables for multivariate analysis, losing the statistical significance of this variable. In the literature, a study found divergent results on the association of placental abruption and fetal or neonatal death [13]. Another investigation showed that neonates born to mothers who had complications during delivery were more likely to die [28]. A similar result was found in other research that showed significant associations between perinatal causes (resulting from complications of pregnancy, labor and delivery) and neonatal death [29].

Recent Brazilian systematic review and metanalysis about risk factors related to pregnant women (absence of partners, age over 35 years old, twin pregnancy absence or inadequate prenatal care, presence of complications during pregnancy and cesarean delivery) and to the neonate (prematurity, low birth weight, congenital malformations and perinatal anoxia) showed associations between those and neonatal deaths [30].

These results were like the ones found in the final analysis of this research concerning the variable perinatal asphyxia.

The described cases had shown a strong association between perinatal anoxia and neonatal death (RR = 2.669 (95% CI = 1.48–4.813), *p* < 0.001), reassuring findings from other studies [4,22,30,31]. The perinatal asphyxia index reflects the quality of care not only for a newborn but also for entire gestational period [5].

In this research, early neonatal sepsis was the most strongly associated with the outcome and presented high relative risk for death (RR = 6.843 (95% CI = 1.824–25.675), *p* < 0.004). The North and Northeast Brazilian regions had the highest proportions of neonatal deaths caused by infections (20.7% and 26.9%, respectively) [22], confirming the results of the current study.

On the other hand, an ecological time series study, carried out with records of neonatal deaths from 2000 to 2018 through the Mortality Information System, revealed a trend toward a reduction in preventable early and late neonatal mortality in the Brazilian regions. This study identified the most frequent causes of preventable neonatal mortality, which were birth asphyxia, respiratory distress syndrome and septicemia [32].

A study carried out in Cuiabá city observed that among neonates who died in the first week of life, most required umbilical catheterization in the NICU, but this was not significantly related to early neonatal deaths [33]. In this research, however, umbilical venous catheterization was performed in 84.6% of neonates who died. This was significantly associated with death in the bivariate analysis of the sample and in the multivariate analysis in the final model, and venous umbilical catheterization can be an indicator of severity of illness or sepsis [34].

The NMRs of this cohort (3.47 deaths per thousand LBs (95% CI = 1.10–8.03 per thousand LBs)) and the early and late neonatal components and the postneonatal mortality rates found in the evaluated populations (2.35 deaths per thousand LBs (95% CI = 0.95–7.23 deaths per thousand LBs), 1.12 deaths per thousand LBs (95% CI = 0.08–5.08 deaths per thousand LBs) and 0.51 postneonatal deaths per thousand LBs (95% CI = 0.05–2.25 deaths per thousand LBs), respectively), were below Brazil’s national average in 2018 (9.1 deaths per thousand LBs) [35] and slightly lower but nonsignificantly different from the rate of Santa Catarina (6.9 deaths per thousand LBs), the state with the lowest mortality rate between Brazil’s state capitals in the same period [4]. The results presented demonstrate that the rates of early neonatal death were like those of a cohort study carried out in a private hospital [27] and to those obtained in international studies [14]. It is important to bear in mind that the present study excluded twins, neonates with malformations incompatible with life and babies transferred to and from other hospitals or readmitted to the NICU, all of which are very rare but could have the effect of altering the comparison conditions.

These results can be credited to the quality of care provided in the Obstetrics and Neonatology Services of the hospital where this study was carried out. This leads to a reflection on the difficulties in accessing reproductive health, as well as on the quality of health and education actions for the population within the Public Health System (Sistema Único de Saúde, SUS) [18], but also leaves space to understand where to improve in order to reduce the (neonatal) mortality rate.

The Brazilian Society of Pediatrics, through the Neonatal Resuscitation Program (NRP), has contributed greatly to the reduction in neonatal morbidity and mortality associated with perinatal asphyxia throughout Brazil [36]. The NRP consists of training in resuscitation practices in the delivery room, based on documents published by the International Liaison Committee on Resuscitation—Neonatal Life Support Task Force [37,38], which serve as a guide adapted to the reality of each country [36].

## 5. Conclusions

This study found very low neonatal and postneonatal mortality, consistent with the data from the international literature, which can be credited to the quality of care available in prenatal care, delivery and the NICU. It is noteworthy that these pregnant women from different social classes had in common a health plan with direct access to health services, which provided them with excellent care throughout their pregnancy and postpartum period.

Even so, a significant association was found between deaths and the determinants—history of abortion, perinatal asphyxia, neonatal infection and umbilical venous catheterization—which are preventable causes. The findings show that there are possibly gaps in the care provided to these pregnant women and the newborns, which will be analyzed by the institution’s managers.

These results also indicate to governments that the reduction of neonatal mortality and child mortality is possible through the adoption of strategies that promote improved access to health services and the quality of care for pregnant women and newborns. These measures may be effective in addition to an eventual expansion of the population coverage of sanitation and drinking water.

## Figures and Tables

**Table 1:** Descriptive and bivariate analyzes of environmental and maternal variables according to the theoretical model, studied in the cohort from 2013 to 2018, in Fortaleza (CE), Brazil (n = 480).

Variable (n = 480) Distribution by Blocks	All n (%)	Death		*p*-Value *	RR	95% CI
		Yes n (%)	No n (%)			
**Block 1—Maternal sociodemographic characteristics and environmental factors**						
Age (years) ^a^	31.2 ± 5.70; 13–58	31.0 ± 5.99	31.2 ± 5.65	0.895	0.996	0.943–1.030
>35	105 (21.9)	11 (28.2)	94 (21.3)	0.316	1.450	0.696–3.021
≤35	375 (78.1)	28 (71.8)	347 (78.7)		1.000	-
Marital status						
Single/without stable union	117 (24.4)	10 (25.6)	107 (24.3)	0.847	1.076	0.508–2.281
Married/stable union	363 (75.6)	29 (74.4)	334 (75.7)		1.000	-
Education						
Elementary (I and II)	19(4.0)	0(0.0)	19(4.3)	0.566	-	-
Medium	148(30.8)	15(38.5)	133(30.2)		-	-
Incomplete college	70(14.6)	5(12.8)	65(14.7)		-	-
Complete college	243(50.6)	19(48.7)	224(50.8)		-	-
Education: college—complete/incomplete						
YES	313 (65.2)	24 (61.5)	289 (65.5)	0.604	1.000	-
NO	167 (34.8)	15 (38.5)	152 (34.5)		1.171	0.632–2.171
Potable water						
YES	471 (98.1)	38 (97.4)	433 (98.2)	0.537	-	-
NO	9 (1.9)	1 (2.6)	8 (1.8)		-	-
Installed sewage system						
YES	247 (51.5)	19 (48.7)	228 (51.7)	0.741	1.000	-
NO	233 (48.5)	20 (51.3)	213 (48.3)		1.116	0.611–2.037
Potable water + sewage system						
YES	247 (51.5)	19 (48.7)	228 (51.7)	0.741	1.000	-
NO	233 (48.5)	20 (51.3)	213 (48.3)		1.116	0.611–2.037
**Block 2—Pregnancy and associated comorbidities**						
Prenatal consultation ^a^	6.8 ± 2.28;0–15	6.3 ± 2.33	6.9 ± 2.27	**0.121**	0.894	0.776–1.030
Pregnancy number ^a^	1.8 ± 1.06;1–8	1.9 ± 1.22	1.7 ± 1.05	0.448	1.105	0.854–1.429
1	261 (54.4)	20 (51.3)	241 (54.7)	0.389	1.000	-
2	134 (27.9)	9 (23.1)	125 (28.3)		0.876	0.410–1.872
≥ 3	85 (17.7)	10 (25.6)	75 (17.0)		1.535	0.748–3.150
Parity ^a^	1.4 ± 0.69; 1–5	1.4 ± 0.68	1.4 ± 0.69	0.924	0.979	0.628–1.25
1	309 (64.4)	25 (64.1)	284 (64.4)	0.927	1.375	0.341–5.556
2	137 (28.5)	12 (30.8)	125 (28.3)		1.489	0.350–6.342
≥ 3	34 (7.1)	2 (5.1)	32 (7.3)		1.000	-
Abortion history						
YES	106 (22.1)	14 (35.9)	92 (20.9)	**0.042**	1.976	1.062–3.664
NO	374 (77.9)	25 (64.1)	349 (79.1)		1.000	-
Antenatal corticosteroids ^b^ (n = 362)						
YES	149 (41.2)	8 (34.8)	141 (41.6)	0.663	0.762	0.332–1.752
NO	213 (58.8)	15 (65.2)	198 (58.4)		1.000	-
Presentation						
Cephalic	57 (95.0)	32 (82.1)	388 (88.0)	0.309	0.653	0.302–1.413
Pelvic/feet/breech	3 (5.0)	7 (17.9)	53 (12.0)		1.000	-
Eclampsia						
YES	8 (1.7)	2 (5.1)	6 (1.4)	**0.132**	3.189	0.923–1.015
NO	472 (98.3)	37 (94.9)	435 (98.6)		1.000	-
HELLP Syndrome						
YES	13 (2.7)	1 (2.6)	12 (2.7)	1.000	0.945	0.14–6.369
NO	467 (97.3)	38 (97.4)	429 (97.3)		1.000	-
Preeclampsia/chronic high blood pressure						
YES	110 (22.9)	11 (28.2)	99 (22.4)	0.428	1.321	0.680–2.567
NO	370 (77.1)	28 (71.8)	342 (77.6)		1.000	-
Hypertensive syndromes						
YES	124 (25.8)	13 (33.3)	111 (25.2)	0.258	1.435	0.762–2.705
NO	356 (74.2)	26 (66.7)	330 (74.8)		1.000	-
Gestational diabetes						
YES	38 (7.9)	1 (2.6)	37 (8.4)	0.348	0.306	0.043–2.168
NO	442 (92.1)	38 (97.4)	404 (91.6)		1.000	-
Third-trimester bleeding						
YES	30 (6.2)	5 (12.8)	25 (5.7)	**0.085**	2.206	0.931–5.228
NO	450 (93.8)	34 (87.2)	416 (94.3)		1.000	-
Thrombophilia						
YES	14 (2.9)	0 (0.0)	14 (3.2)	0.617	-	-
NO	466 (97.1)	39 (100.0)	427 (96.8)		-	-
Urinary tract infection						
YES	86 (17.9)	4 (10.3)	82 (18.6)	0.275	0.524	0.191–1.434
NO	394 (82.1)	35 (89.7)	359 (81.4)		1.000	-
TORCHS ^c^						
Present	13 (2.7)	1 (2.6)	12 (2.7)	1.000	0.945	0.14–6.369
Absent	467 (97.3)	38 (97.4)	429 (97.3)		1.000	-
Complications in labor						
YES	191 (39.8)	23 (59.0)	168 (38.1)	**0.016**	2.175	1.180–4.008
NO	289 (60.2)	16 (41.0)	273 (61.9)		1.000	-
Delivery type						
Cesarean	414 (86.2)	31 (79.5)	383 (86.8)	0.223	0.618	0.297–1.285
Vaginal	66 (13.8)	8 (20.5)	58 (13.2)		1.000	-

^a^ Variable described as mean ± standard deviation; as minimum–maximum value and by category. ^b^ Indicated at 24 to 36 weeks and 6 days of gestation. ^c^ TORCHS: toxoplasmosis, rubella, cytomegalovirus, herpes simplex and syphilis; *p*-value *: Fisher’s or Pearson’s chi-square test (categorical variables) and Mann–Whitney test (quantitative variables). Bold *p*-values stand for significant effects.

**Table 2:** Descriptive and bivariate analyzes of newborn variables according to the theoretical model, studied in the cohort from 2013 to 2018 in Fortaleza (CE), Brazil (n = 480).

Variable (n = 480) Distribution by Blocks	All n (%)	Death		*p*-Value *	RR	95% CI
		Yes n (%)	No n (%)			
**Block 3—Characteristics of the newborn**						
Sex						
Male	273 (56.9)	25 (64.1)	248 (56.2)	0.401	1.354	0.722–2.539
Female	207 (43.1)	14 (35.9)	193 (43.8)		1.000	-
Gestational age (weeks) ^a^	33.8 ± 3.93; 22–41	30.3 ± 5.97	34.1 ± 3.54	**<0.001**	0.831	0.772–0.894
Premature	369 (76.9)	29 (74.4)	340 (77.1)	0.694	0.872	0.439–1.734
At term	111 (23.1)	10 (25.6)	101 (22.9)		1.000	-
Birth weight (grams) ^a^	2220 ± 890; 360–5060	1610 ± 1120	2280 ± 850	**0.001**	0.427	0.259–0.704
Birth weight class (grams) <1500	109 (22.7)	23 (59.0)	86 (19.5)	**<0.001**	4.893	2.682–8.927
≥1500	371 (77.3)	16 (41.0)	355 (80.5)		1.000	-
Birth weight class (grams) <2500	289 (60.2)	27 (69.2)	262 (59.4)	0.306	1.487	0.772–2.863
≥2500	191 (39.8)	12 (30.8)	179 (40.6)		1.000	-
Birth weight adequacy by GA ^b^ (WHO) ^c^ (n = 473)						
SGA ^d^	138 (29.2)	15 (38.5)	123 (28.3)	0.199	1.580	0.802–3.113
AGA ^e^/LGA ^f^	335 (70.8)	24 (61.5)	311 (71.7)		1.000	-
Apgar score, 1st minute ^a^	7.0 ± 2.08; 0–10	5.1 ± 2.77	7.2 ± 1.92	**<0.001**	0.727	0.654–0.809
With anoxia (Apgar < 7)	203 (42.3)	28 (71.8)	175 (39.7)	**<0.001**	8.599	4.192–7.637
No anoxia (Apgar 7–10)	277 (57.7)	11 (28.2)	266 (60.3)		1.000	-
Apgar score, 5th minute ^a^	8.4 ± 1.2; 1–10	7.05 ± 2.03	8.52 ± 1.02	**<0.001**	0.641	0.565–0.728
With anoxia (Apgar < 7)	69 (14.4)	19 (48.7)	50 (11.3)	**<0.001**	5.659	3.188–10.044
No anoxia (Apgar 7–10)	411 (85.6)	20 (51.3)	391 (88.7)		1.000	-
**Block 4—Clinical complications of the newborn and therapeutic interventions**						
Resuscitation						
YES	173 (36.0)	28 (71.8)	145 (32.9)	**<0.001**	4.517	2.307–8.846
NO	307 (64.0)	11 (28.2)	296 (67.1)		1.000	-
Perinatal asphyxia						
YES	42 (8.8)	14 (35.9)	28 (6.3)	**<0.001**	5.840	3.294–10.353
NO	438 (91.2)	25 (64.1)	413 (93.7)		1.000	-
Respiratory Distress Syndrome						
YES	211 (44.0)	27 (69.2)	184 (41.7)	**0.001**	2.868	1.489–5.526
NO	269 (56.0)	12 (30.8)	257 (58.3		1.000	-
Early neonatal sepsis						
YES	212 (44.2)	36 (92.3)	176 (39.9)	**<0.001**	15.170	4.737–48.581
NO	268 (55.8)	3 (7.7)	265 (60.1)		1.000	-
Late neonatal sepsis						
YES	43 (9.0)	5 (12.8)	38(8.6)	0.377	1.495	0.617–3.621
NO	437 (91.0)	34 (87.2)	403 (91.4)		1.000	-
Oxygen (days) ^a,g^ (n = 452)	9.7 ± 20.55; 1–159	12.4 ± 24.57	9.5 ± 20.15	0.418	1.005	0.993–1.018
YES	452 (94.2)	39 (100.0)	413 (93.7)	0.153	-	-
NO	28 (5.8)	0 (0.0)	28 (6.3)		-	-
Mechanical ventilation (days) ^a,g^ (n = 154)	9.0 ± 15; 1–102	10.3 ± 19.49	8.6 ± 13.42	0.607	1.005	0.986–1.024
YES	154 (32.1)	35 (89.7)	119 (27.0)	**<0.001**	18.523	6.702–51.191
NO	326 (67.9)	4 (10.3)	322 (73.0)		1.000	-
Parenteral nutrition (days) ^a,g^ (n = 129)	9.3 ± 9.28; 1–70	10.0 ± 16.64	9.1 ± 6.33	0.676	1.008	0.972–1.045
YES	129 (26.9)	26 (66.7)	103 (23.4)	**<0.001**	5.442	2.885–10.263
NO	351 (73.1)	13 (33.3)	338 (76.6)		1.000	-
Venous umbilical catheter (days) ^a,g^ (n = 174)		6.1 ± 3.83; 1–38	4.2 ± 3.89	6.6 ± 3.7	**<0.001**	0.808
YES	174 (36.2)	33 (84.6)	141 (32.0)	**<0.001**	9.672	4.135–22.624
NO	306 (63.8)	6 (15.4)	300 (68.0)		1.000	-
Peripherally inserted central catheter (days) ^a,g^ (n = 83)	19.7 ± 15.72; 1–65	15.9 ± 14.61	20.2 ± 15.89	0.470	0.981	0.932–1.033
YES	86 (17.9)	10 (25.6)	76 (17.2)	0.194	1.580	0.800–3.118
NO	394 (82.1)	29 (74.4)	365 (82.8)		1.000	-
Venous dissection						
YES	6 (1.2)	3 (7.7)	3 (0.7)	**0.008**	6.583	2.787–15.551
NO	474 (98.8)	36 (92.3)	438 (99.3)		1.000	-
Hospitalization (days) ^a^	18.8 ± 24.77; 1–171	14.8 ± 24.5	19.1 ± 24.79	0.317	0.992	0.975–1.008

^a^ Variable described in mean ± standard deviation and minimum value–maximum value; ^b^ gestational age; ^c^ World Health Organization; ^d^ small for gestational age; ^e^ adequate for gestational age; ^f^ large for gestational age; ^g^ among those who performed; *p*-value *: Fisher’s test or Pearson’s chi-square (categorical variables) and Mann–Whitney test (quantitative variables). Bold *p*-values stand for significant effects.

**Table 3:** Bivariate analysis (*p* = 0.15)/multivariate analysis by blocks/multivariate analysis—final model (*p* < 0.05) of the variables studied in the cohort in Fortaleza (CE), Brazil (n = 480).

	Analysis of Newborn Deaths								
	Bivariate			Multivariate per Block			Final Multivariate		
Variables	*p*-Value *	RR	95% CI	*p*-Value **	RR	95% CI	*p*-Value **	RR	95% CI
**Block 2—Pregnancy and associated comorbidities**									
Prenatal consultation	0.121	0.894	0.776–1.030	-	-	-	-	-	-
Abortion history (YES)	**0.042**	1.976	1.062–3.664	**0.019**	2.084	1.131–3.841	**0.047**	1.774	1.007–3.126
Antenatal corticosteroids ^a^ (YES)	0.663	0.762	0.332–1.752	-	-	-	-	-	-
Eclampsia (YES)	0.132	3.189	0.923–11.015	-	-	-	-	-	-
Third-trimester bleeding (YES)	0.085	2.206	0.931–5.228	-	-	-	-	-	-
Complications in labor (YES)	**0.016**	2.175	1.180–4.008	**0.008**	2.263	1.234–4.148	-	-	-
**Block 3—Characteristics of the newborn**									
Gestational age (weeks)	**<0.001**	0.831	0.772–0.894	**0.001**	0.890	0.831–0.953	-	-	-
Birth weight (grams) <1500	**<0.001**	4.893	2.682–8.927	**0.024**	1.924	1.091–3.394	-	-	-
Apgar score, 1st minute	**<0.001**	0.727	0.654–0.809	**<0.001**	0.800	0.717–0.893	-	-	-
Apgar score, 5th minute: with anoxia (Apgar < 7)	**<0.001**	5.659	3.188–10.044	-	-	-	-	-	-
**Block 4—Clinical complications of the newborn and therapeutic interventions**									
Resuscitation (YES)	**<0.001**	4.517	2.307–8.846	-	-	-	-	-	-
Perinatal asphyxia (YES)	**<0.001**	5.840	3.294–10.353	**0.001**	2.655	1.469–4.800	**0.001**	2.669	1.480–4.813
Respiratory Distress Syndrome (YES)	**0.001**	2.868	1.489–5.526	-	-	-	**-**	-	-
Early neonatal sepsis (YES)	**<0.001**	15.170	4.737–48.581	**0.005**	6.841	1.789–26.164	**0.004**	6.843	1.824–25.675
Mechanical ventilation (YES)	**<0.001**	18.523	6.702–51.191	-	-	-	-	-	-
Parenteral nutrition (YES)	**<0.001**	5.442	2.885–10.263	-	-	-	-	-	-
Venous umbilical catheter (YES)	**<0.001**	9.672	4.135–22.624	**0.018**	3.359	1.227–9.196	**0.021**	3.275	1.199–8.948
Time of venous umbilical catheter (n = 174)	**0.001**	0.808	0.711–0.919	-	-	-	**-**	-	-
Venous dissection (YES)	**0.033**	6.583	2.353- 8.423	-	-	-	**-**	-	-

^a^ Gestational age between 24 and 36 weeks; *p*-value *: Fisher’s test or Pearson’s chi-square (categorical variables) and Mann–Whitney test (quantitative variables); *p*-value **: Poisson’s regression. Bold *p*-values stand for significant effects.

## Data Availability

The data presented in this study are available on reasonable request from the corresponding author, although the raw data will not be shared due to the privacy restrictions of the participants.
